# Raman Spectroscopic Characterization of Polymerization Kinetics of Cyanoacrylate Embolic Glues for Vascular Embolization

**DOI:** 10.3390/polym13193362

**Published:** 2021-09-30

**Authors:** Yongjiang Li, Lei Xiao, Zian Wang, Kejie Chen, Chundong Xue, Miao Yu, Yu Wang, Fanyi Kong, Kun Liu, Kairong Qin

**Affiliations:** 1 School of Optoelectronic Engineering and Instrumentation Science, Dalian University of Technology, Dalian 116024, China; yongjiangli@dlut.edu.cn (Y.L.); 31942035@mail.dlut.edu.cn (L.X.); wangza@mail.dlut.edu.cn (Z.W.); ckj@dlut.edu.cn (K.C.); xuechundong@dlut.edu.cn (C.X.); yuwang0410@dlut.edu.cn (Y.W.); kongfanyi@mail.dlut.edu.cn (F.K.); 2 School of Biomedical Engineering, Dalian University of Technology, Dalian 116024, China; yumiao@mail.dlut.edu.cn

**Keywords:** polymerization kinetics, Raman spectroscopy, cyanoacrylate glues, endovascular embolization

## Abstract

Endovascular glue embolization is a minimally invasive technique used to selectively reduce or block the blood supply to specific targeted vessels. Cyanoacrylate glues, mixed with radiopaque iodized oil, have been widely used for vascular embolization owing to their rapid polymerization rate, good penetration ability and low tissue toxicity. Nevertheless, in clinical practice, the selection of the glue–oil proportion and the manual injection process of mixtures are mostly based on empirical knowledge of operators, as the crucial physicochemical effect of polymerization kinetics has rarely been quantitatively investigated. In this study, the Raman spectroscopy is used for studying the polymerization kinetics of n-butyl-cyanoacrylate-based glues mixed with an iodized oil. To simulate the polymerization process during embolization, glue–oil mixtures upon contact with a protein ionic solution mimicking blood plasma are manually constructed and their polymerization kinetics are systematically characterized by Raman spectroscopy. The results demonstrate the feasibility of Raman spectroscopy in the characterization of polymerization kinetics of cyanoacrylate-based embolic glues. The polymerization process of cyanoacrylate-based mixtures consists of a fast polymerization phase followed by a slow phase. The propagation velocity and polymerization time primarily depend on the glue concentrations. The commonly used 50% mixture polymerizes 1 mm over ∼21.8 s, while it takes ∼51 min to extend to 5 mm. The results provide essential information for interventional radiologists to help them understand the polymerization kinetics of embolic glues and thus regulate the polymerization rate for effective embolization.

## 1. Introduction

**Figure 1:**
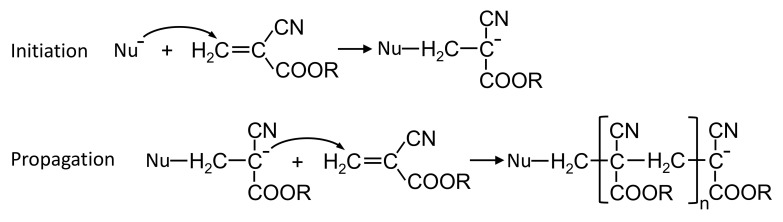
Initiation and propagation steps for alkyl (R) cyanoacrylate polymerization activated by nucleophile (Nu${}^{-}$).

Endovascular embolization with embolic glues is a minimally invasive therapeutic technique used to selectively reduce or block the blood supply to specific targeted sites of the body. It is carried out under X-ray by delivering embolic glues into the circulation through a microcatheter. This technique has been established as an adjuvant treatment strategy to surgical therapies. With the development in embolic agents and endovascular techniques, embolization with embolic glues gradually transfers from an adjunctive to a definitive treatment modality in the management of arteriovenous malformations, tumors, trauma or hemorrhage. Due to the advantages of minimal invasion, permanent embolizaiton and low inflammation, this technique continuously expands its roles with new applications regularly appearing in medical practice [1,2,3]. N-butyl cyanoacrylate (NBCA) glues are commonly used liquid embolic agents for the occlusion of tortuous and intertwined vascular malformations owing to their low viscosity, excellent permeability, rapid polymerization rate and low tissue toxicity [4,5]. The occlusion mechanism of NBCA glues is the activation of anionic and zwitterionic polymerization induced by anions, amino acids and nucleophilic compounds in the blood plasma (Figure 1) [6,7,8].

In clinical practice, NBCA embolic glues are usually mixed with iodized oil (Lipiodol Ultra Fluid, Laboratoire Guerbet, France) to adjust the polymerization rate and enable the radio-opacity. Upon injection into the blood flow, glue–oil mixture immediately polymerizes in the presence of anions and amino acids in the plasma, forming a soft-shell droplet. Simultaneously, the emerging droplet flows and permeates with the blood steam to targeted vessels, resulting in a solidified cast for complete vascular occlusions. During the injection and occlusion processes, the polymerization kinetics play an essential role in the control of droplet structure and subsequent distribution [9,10,11]. High glue concentration mixtures (e.g., pure glue or $C_{G}\geq$ 75%) tend to occlude towards high-flow vessels and proximal arteries, while low concentration mixtures (e.g., $C_{G}∼$ 25%) permeate further for the occlusion of distal niduses and small low-flow vessels [12,13,14]. Yet glue mixtures of high concentrations (e.g., $C_{G}\geq$ 50%) can cause the adhesion of the microcatheter to the blood vessels [15,16]. Low glue concentration mixtures have the risks of penetration into critical vein branches, leading to non-targeted occlusion [17,18]. To successfully deliver and block targeted vessels, the polymerization rate relevant to glue concentration should be optimized. However, clinically, the concentration of glue mixture and its injection rate are adjusted empirically by estimating the transit time for the contrast medium to reach the lesion, showing a great dependence on training and experience of operators [3]. Little quantitative information exists in regard to the polymerization kinetics of glue–oil mixtures.

Extensive works [6,7,8,19] have been carried out to study the polymerization kinetics of pure cyanoacrylate glues. The polymerization is considered to be triggered by nucleophiles leading to anionic or zwitterionic polymerization (Figure 1). Some previous studies have measured the polymerization time of NBCA glues mixed with iodized oil [20,21,22,23,24]. The polymerization time is primarily evaluated by dropping NBCA/iodized oil mixture at different ratios onto blood plasma or tissue samples [20,21,22,23,24]. The changes in polymer morphology or light transmittance are recorded with high-speed camera for characterizing the polymerization time. However, the change in morphology or light transmittance depends on the volume of deposited mixture and the ending criteria of polymerization are not precisely defined. Recently, a novel experimental setup has been proposed to characterize polymerization kinetics of glue–oil mixtures in contact with various blood substitutes [9,10,11]. The polymerization process of glue–oil mixtures and the corresponding polymerization rates have been systematically investigated. Nevertheless, this technique determines the polymerization kinetics through the opacity change due to density variation during polymerization. It is still challenging to correlate the physical change with its evolution of chemical reactions during polymerization. Raman spectroscopy is a modern spectroscopic method with a potential to determine chemical details of molecular structure, which is non-invasive, non-destructive and usually contact-free. This technique has been recently applied to study the polymerization of cyanoacrylate-based glues [25,26,27,28,29,30]. Numerous studies have employed Raman spectroscopy to study curing of cyanoacrylate-fumed fingerprints and the kinetics of cyanoacrylate-based adhesives, as well as their dependences on substrate types, temperature and film thickness [25,26,27,28,29,30]. Recently, Nedvedova et al. [12] analyzed the kinetics of n-Butyl-cyanoacrylate adhesives and its oily mixtures using Raman spectroscopy by contacting drops of two reaction solutions. The polymerization creation method allows for the characterization of polymerization of a thin film, which cannot reflect the volumetric polymerization of glue–oil mixtures. Besides, the effects of glue concentration and blood composition are rarely investigated.

The objectives of the present study are to characterize the polymerization kinetics of NBCA/oil mixtures in contact with blood substitutes using the Raman spectroscopy. Firstly, the polymerization of glue/oil mixtures in volumetric bulk during vascular embolization is finely modeled. Then, the polymerization kinetics of glue/oil mixture upon contact with a blood substitute is measured experimentally using Raman spectroscopy. The polymerization process is characterized and the polymerization rate is evaluated. Moreover, the dependence of polymerization kinetics on glue concentration and blood composition are systematically studied.

## 2. Materials and Methods

### 2.1. Raman Spectroscopy

**Figure 2:**
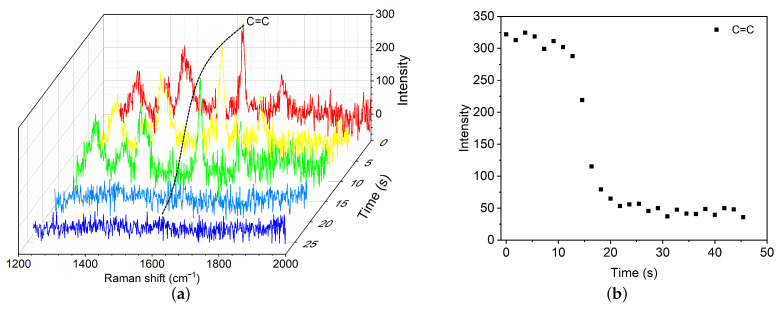
Schematic of experiment setup for characterizing the polymerization kinetics of glue–oil mixtures induced by blood substitute.

Raman spectroscopy enables chemical identification of elements and compounds during polymerization of glue mixture activated by blood substitute. In the study, the time-evolution of spectra during polymerization is captured using a Renishaw’s InVia Raman microscope (H25717, Renishaw, UK) equipped with a charge-coupled device (CCD) camera (Figure 2). The spectrometer is fitted with rayleigh rejection filters and gratings. The Raman microscope is equipped with a microscope (Carl Zeiss, Germany) with three objectives (×4, ×50 and ×100), which accommodate the CCD camera allowing capture of spectra. The spectrometer is controlled by PC with instrument-control software (Renishaw WiRE 3.4). Sample excitation is achieved using a laser at excitation wavelengths of either 532 nm, 633 nm and 785 nm, which is controlled by a laser system. To eliminate the thermal effect on polymerization, the 532 nm laser is applied in this study. Under 532 nm excitation, Raman shift within the range of 1500–3200 cm${}^{-1}$ can be measure with a spectral resolution of 1 cm${}^{-1}$.

### 2.2. Sample Preparation

To characterize the polymerization kinetics of embolic glue mixed with iodized oil, an N-butyl cyanoacrylate-based embolic glue (Fuaile, Beijing, China) is used in the study. Similar to clinical practice, an iodized oil registered as Iodinated Oil Injection (Luyin, Yantai, China) is mixed with the embolic glue to regulate its polymerization rate. The embolic glue is stored in sealed containers at 4 ${}^{\circ }$C and left at room temperature 5 min before use. The iodized oil is, however, stored at room temperature. A 0.2 mL total volume of glue and iodized oil is mixed with volume ratios 1:0, 2:1, 1:1 and 1:2, corresponding to glue concentrations $C_{G}$ 100%, 67%, 50% and 33%, respectively. The mixing process consists of manually passing the liquids from one syringe to the other several times at a high frequency (about 150 times in 90 s). All the experiments are conducted at room temperature of 20 ${}^{\circ }$C.

During clinical embolization, the polymerization of glue–oil (GO) mixtures is activated by blood. To study the blood components on polymerization, blood substitutes are prepared manually according to the composition of physiological blood plasma. Blood naturally consists of a suspension of cells in plasma, which is made of proteins (∼6–8%), metallic ions (∼1%) and water (∼91–93%). A substitute of blood plasma is prepared by mixing phosphate buffer saline with Bovine albumin (Polygene, Fuzhou, China), referred to as PBS and BSA respectively. The PBS contains the main blood ions in the same proportion as plasma. BSA is incorporated with two different concentrations: 40 g/L and 80 g/L. The resulting solutions are respectively denoted BSA4 and BSA8 in the following. Note that BSA8 can be considered as a model of blood plasma where all the proteins are represented by BSA, whereas BSA4 allows us to evaluate the role of albumin only.

### 2.3. Experimental Procedure

To model the polymerization of injected GO mixture bulk upon contact with blood, the polymerization reaction is created using a glass capillary tube (internal diameter $D_{t}=$ 1.0 mm). As shown in Figure 2, a volume of GO mixture is first aspirated into the glass tube by means of a syringe connected to the tube end. The tube containing GO mixture is fixed horizontally on a glass slide, which is placed on the stage of microscope. Then, a drop of blood substitute is dripped at the tube tip to start up the polymerization reaction (black box in Figure 2).

As described in our previous study [9,11], upon contact with a a proteinaceous solution, the polymerization of GO mixture proceeds so fast. The polymerization consists of a fast volumetric polymerization phase followed by a slow phase. In the fast phase, the polymerization propagates ∼2.1 mm over an average time of ∼132 s. In the slow phase, the polymerization front propagates slowly until the end of the mixture. Because of this, the Raman spectra in the fast phase is measured at location 1 mm to the tube tip, which is defined as the origin z=0. The evolution of C=C intensity at ∼1617 cm${}^{-1}$ is used to characterize the polymerization rate. To speed up the sampling rate, Raman shift is set within the narrow-band of C=C (1245–2030 cm${}^{-1}$). In the slow phase, the Raman spectra is measured at location z=5 mm within 1500–3200 cm${}^{-1}$.

## 3. Results

### 3.1. Raman Spectroscopic Characterization of Polymerization Process

#### 3.1.1. Characterization of Fast Polymerization Phase

**Figure 3:**
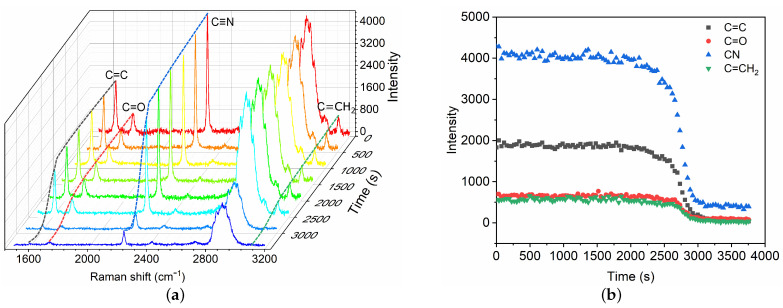
Time evolution of Raman spectra (**a**) and C=C intensity change at 1617 cm${}^{-1}$ (**b**) during the fast polymerization phase of GO mixture ($C_{G}=50\%$) in contact with blood substitute BSA8.

To characterize the polymerization kinetics of GO mixture, typical results are presented for commonly used GO mixture ($C_{G}=50\%$) in clinic upon contact with BSA8, which is considered as a model of blood plasma. As soon as BSA8 is dripped to contact the GO mixture, a fast phase of polymerization is first activated near the end of GO mixture. The fractional Raman spectra within the narrow-band of C=C are recorded (Figure 3a). The time evolution of C=C intensity is shown as a function of time at the location z=1 mm. As the fast polymerization proceeds, the C=C intensity reduces owing to the propagation of polymerization, which further decreases towards an asymptotic value until the fast phase completes. To measure the polymerization rate, we define the characteristic $t_{f}$ time of the fast phase as the time for which the C=C intensity has decreased by 95%. For the case of GO mixture ($C_{G}=50\%$) with BSA8, the polymerization time is ∼21.8 s. The mean propagation velocity is $V_{f}$ ∼ 2.8 mm/min.

#### 3.1.2. Characterization of Slow Polymerization Phase

**Figure 4:**
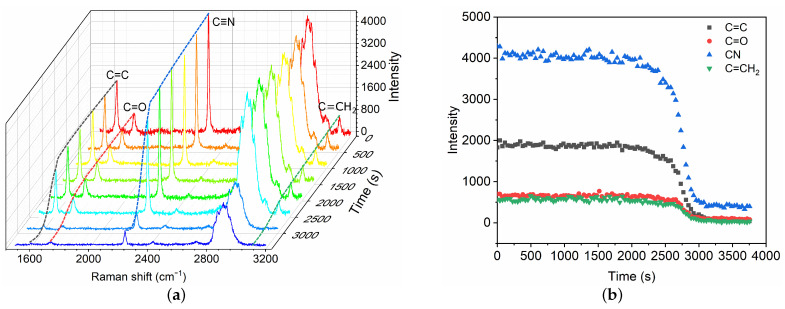
Time evolution of Raman spectra (**a**) and intensity change in chemical bonds (**b**) during the slow polymerization phase of GO mixture in contact with blood substitute BSA8. The Raman shifts of C=C, C=O, C≡N and C=CH${}_{2}$ are 1617 cm${}^{-1}$, 1731 cm${}^{-1}$, 2239 cm${}^{-1}$ and 3128 cm${}^{-1}$, respectively.

After the fast polymerization phase, the polymerization front continues to propagate slowly until the complete mixture has polymerized, which is referred to as the slow volumetric polymerization owing to the characteristic time $t_{s}$. A broadband Raman spectra within 1500–3200 cm${}^{-1}$ are captured (Figure 4) for the characterization of the slow polymerization phase. Intensity changes in typical chemical bonds are measured, including C=C, C=O, C≡N and C=CH${}_{2}$. The variations of different bonds show similar trends. Herein, the C=C intensity is selected as the typical characteristic bond for the measurement of slow polymerization time $t_{s}$. The characteristic $t_{s}$ time is also defined as the time for which the C=C intensity has decreased by 95%. The polymerization time $t_{s}$ is thus ∼51 min in the case, which is asymptotically equal to the time measured by high-speed camera technique [9]. The mean propagation velocity is $V_{s}$ ∼ 0.10 mm/min, which is much lower than that in the fast phase.

### 3.2. Effect of GO Proportion on the Polymerization Process

**Figure 5:**
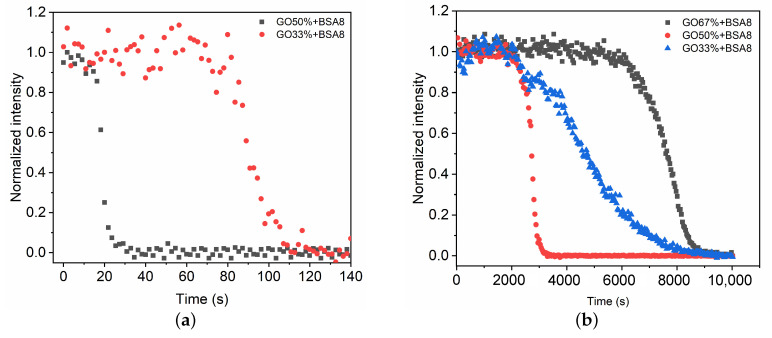
Time evolution of intensity change in C=C during the fast (**a**) and slow (**b**) polymerization phases of GO mixtures in contact with blood substitute BSA8.

We now consider the effect of the glue concentration on the polymerization of a GO mixture in contact with the BSA8 solution. In the case of pure glue ($C_{G}=100\%$) and mixture of high glue proportion ($C_{G}=67\%$), the fast phase polymerizes quickly (∼5 s) and extends over a short distance (∼1 mm). As a result, the Raman spectra during fast phase is difficult to record due to the limitation of sampling time. When the glue concentration decreases below $C_{G}=50\%$, the time evolution of Raman spectra in the fast phase are recorded and C=C intensities at different time intervals are measured (Figure 5a). Compared with the mixture $C_{G}=50\%$, the fast phase for $C_{G}=33\%$ takes longer (∼96.3 s) to extend to the detection point. The mean propagation velocity is $V_{f}$ ∼ 0.6 mm/min. Therefore, the decrease in glue concentration can reduce the polymerization rate and the propagation velocity.

For the slow polymerization phase, the time evolutions of C=C intensity at different glue concentrations are compared (Figure 5b). When the glue concentration is high ($C_{G}=67\%$), it takes more than 2 h to propagate the polymerization to the detection point (z= 5 mm). The polymerization rate and the propagation velocity are slower compared with the mixture $C_{G}=50\%$. This might result from the compact structure formed in the fast phase that prevents the transport of polymerization initiators [9]. As the glue concentration decreases, a relatively fast polymerization rate and propagation velocity occur for the mixture $C_{G}=50\%$. Further decrease in glue concentration $C_{G}=33\%$ leads to the increase in polymerization time $t_{s}=∼$125 min (Table 1).

### 3.3. Effect of Blood Composition on the Polymerization Process

**Figure 6:**
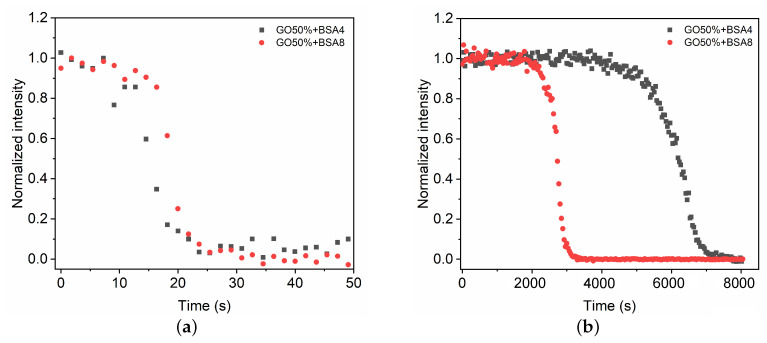
Time evolution of intensity change in C=C during the fast (**a**) and slow (**b**) polymerization phases of the GO mixture $C_{G}=50\%$ in contact with BSA4 and BSA8, respectively.

We now study the effect of the blood composition on the polymerization of the GO mixture $C_{G}=50\%$. BSA4 with half of plasma protein proportion is used to evaluate the role of albumin in polymerization process. As shown in Figure 6, the evolutions of C=C intensities are almost consistent in the fast phase for BSA8 and BSA4. Conversely, in the slow phase low BSA concentration shows a longer polymerization time because of the lower amount of polymerization initiators. This indicates that the BSA concentration of 4% is quite sufficient to induce the fast phase of glue mixture polymerization, while low BSA concentration can reduce the polymerization rate of the slow phase (Table 2).

## 4. Discussion

In this paper, we have demonstrated the application of Raman spectroscopy in the characterization of polymerization kinetics of cyanoacrylate-based glues for vascular embolization. The polymerization reaction is created within a capillary tube which allows for the simulation of the polymerization in volumetric bulk similar to the physiological conditions. The results indicate that Raman spectroscopy is a promising technique in the characterization of polymerization kinetics due to its high specificity of chemical bonds and non-invasive character. We confirm that the polymerization process of GO mixtures induced by protein solution consists of two phases: a fast polymerization (Figure 3) activated by the BSA molecules followed by a slow polymerization phase (Figure 4). In the fast phase, the propagation velocity and polymerization rate is proportional to the glue concentrations (Figure 5a and Table 1). Conversely, polymerization rate in the slow phase depends on both the glue concentration and the BSA concentration.

Considering the practical conditions in clinical endovascular embolization, we have analyzed the influences of GO proportion and blood composition on the polymerization of cyanoacrylate-based embolic glues. For the fast polymerization phase, the polymerization rate primarily depends on the glue concentration, showing no significant difference for BSA4 and BSA8. The results are consistent with our previous study [9]. This finding implies that blood of 4% protein concentration is sufficient to activate fast polymerization phase. Interestingly, in the slow polymerization phase, the largest polymerization rate appears at the medium glue concentration ($C_{G}=50\%$). This phenomenon might be caused by the balance between glue concentration and the number of nucleophilic initiators transported from tube tip to activate polymerization. At high glue concentration ($C_{G}=67\%$), the fast polymerization results in the formation of compact polymer structure [9,30], which prevents the transport of nucleophilic initiators, ultimately leading to a slow polymerization rate. In contrast, at glue concentration of $C_{G}=50\%$, less compact polymer [9] formed in the fast phase facilitates a larger number of initiators being delivered. Despite relatively low glue concentration, glue can polymerize at a high polymerization rate (Table 1).

Conventionally, the polymerization kinetics of cyanoacrylate-based mixtures are characterized by dropping glue mixtures onto blood substitutes or tissue samples [20,21,22,23,24]. The polymerization process is recorded by high-speed camera or video to monitor the morphologic changes. These techniques intuitively present the morphologic evolution owing to the polymerization. However, the mechanism of polymerization is difficult to be inferred from morphologic changes. Compared with the aforementioned techniques, Raman spectroscopy can correlate the polymerization kinetics with the variation of characteristic chemical bonds, which provides essential information for understanding the polymerization mechanism with existing models [31,32]. Although Raman spectroscopy is feasible to characterize polymerization kinetics, there are also limitations. As demonstrated in Section 3.2, the evolution of Raman spectra at high glue concentrations ($C_{G}=67\%$ and $C_{G}=100\%$) is challenging to record due to the high polymerization rate and the low sampling frequency. Even though the narrow-band of C=C is recorded to improve the sampling time, it is still difficult to characterize the fast polymerization phase. Moreover, we have to point out that the polymerization kinetics in this study are investigated at given sampling positions. The results of polymerization time and propagation velocity might not be universal for different sampling positions. These above mentioned limitations result from the coherent characters of Raman spectroscopy, which can be improved by improving Raman spectroscopy techniques. Our previous work has proposed the method of high-speed photography to characterize polymerization kinetics [9,10], allowing the visualization of polymerization process with high spatio-temporal resolution. Alternatively, a comparative study between Raman spectroscopy and high-speed photography methods may be operative to systematically understand the polymerization kinetics.

## 5. Conclusions

In summary, we have established a method to characterize the polymerization kinetics of cyanoacrylate embolic glues using Raman spectroscopy. The results suggest that Raman spectroscopy is a promising technique in the characterization of polymerization kinetics due to its high specificity of chemical bonds and non-invasive character. The polymerization kinetics of cyanoacrylate-based mixtures consist of a fast polymerization phase followed by a slow phase. The propagation velocity and polymerization time greatly depend on the glue concentrations. The commonly used 50% mixture polymerizes 1 mm over ∼21.8 s, while it takes ∼51 min to extend to 5 mm. The feasibility of Raman spectroscopy is limited by high glue concentration for both fast and slow phases. 

## Figures and Tables

**Table 1:** Characteristic polymerization time of fast and slow phases ($t_{f}$ and $t_{s}$) for GO mixtures in contact with BSA8.

Glue Concentration	Blood	Fast Phase $t_{f}$	Slow Phase $t_{s}$
$C_{G}$	Substitute	(z = 1 mm)	(z = 5 mm)
$C_{G}=$ 67%	BSA8	—	8616.8 ± 1145.2 s
$C_{G}=$ 50%	BSA8	21.8 ± 4.3 s	3073.6 ± 531.9 s
$C_{G}=$ 33%	BSA8	96.3 ± 10.2 s	7514.9 ± 883.6 s

**Table 2:** Characteristic polymerization time of fast and slow phases ($t_{f}$ and $t_{s}$) for the GO mixture $C_{G}=50\%$ in contact with BSA4 and BSA8, respectively.

Glue Concentration	Blood	Fast Phase $t_{f}$	Slow Phase $t_{s}$
$C_{G}$	Substitute	(z = 1 mm)	(z = 5 mm)
$C_{G}=$ 50%	BSA4	23.6 ± 4.8 s	6720.1 ± 1088.7 s
$C_{G}=$ 50%	BSA8	21.8 ± 4.3 s	3073.6 ± 531.9 s

## Data Availability

The data presented in this study are available on request from the corresponding author.
